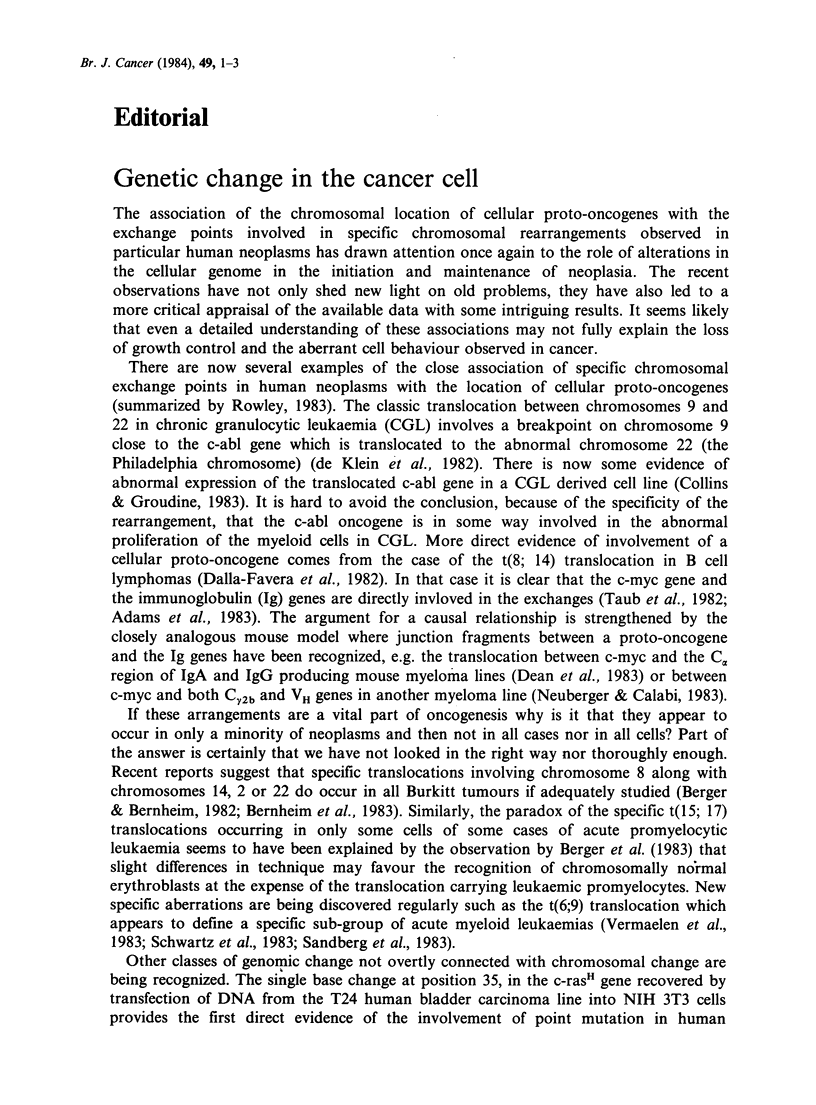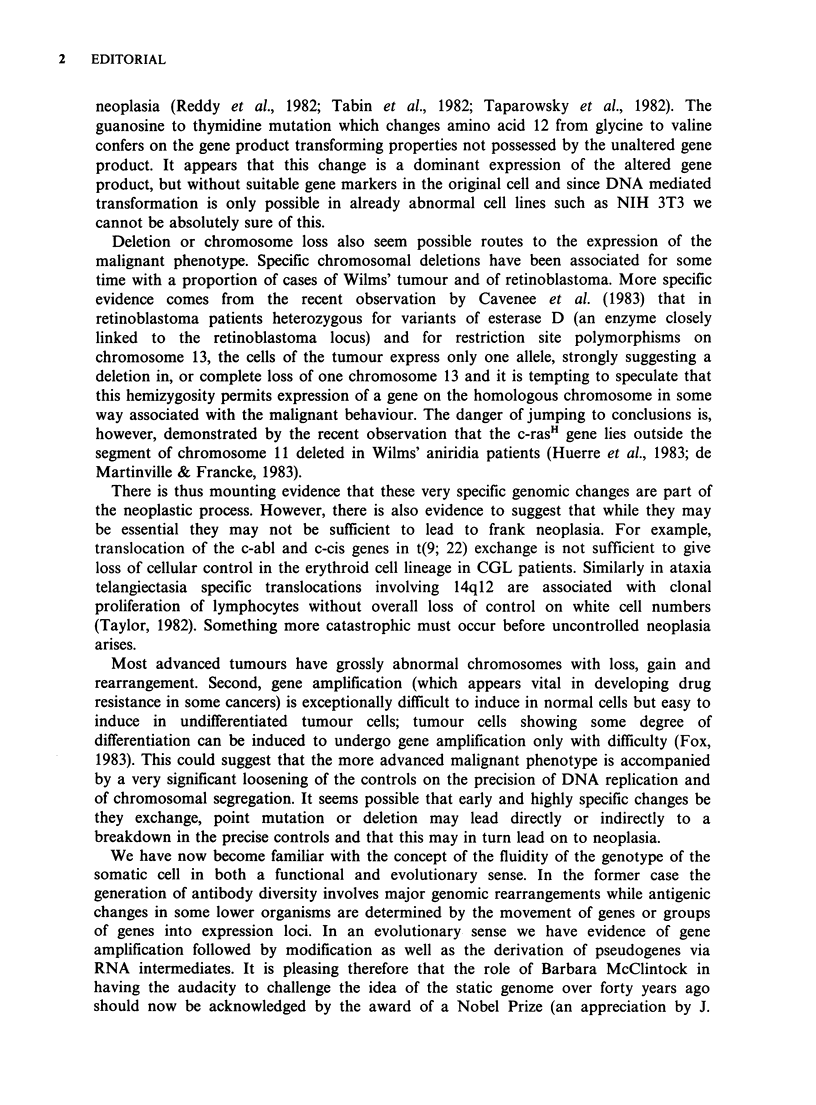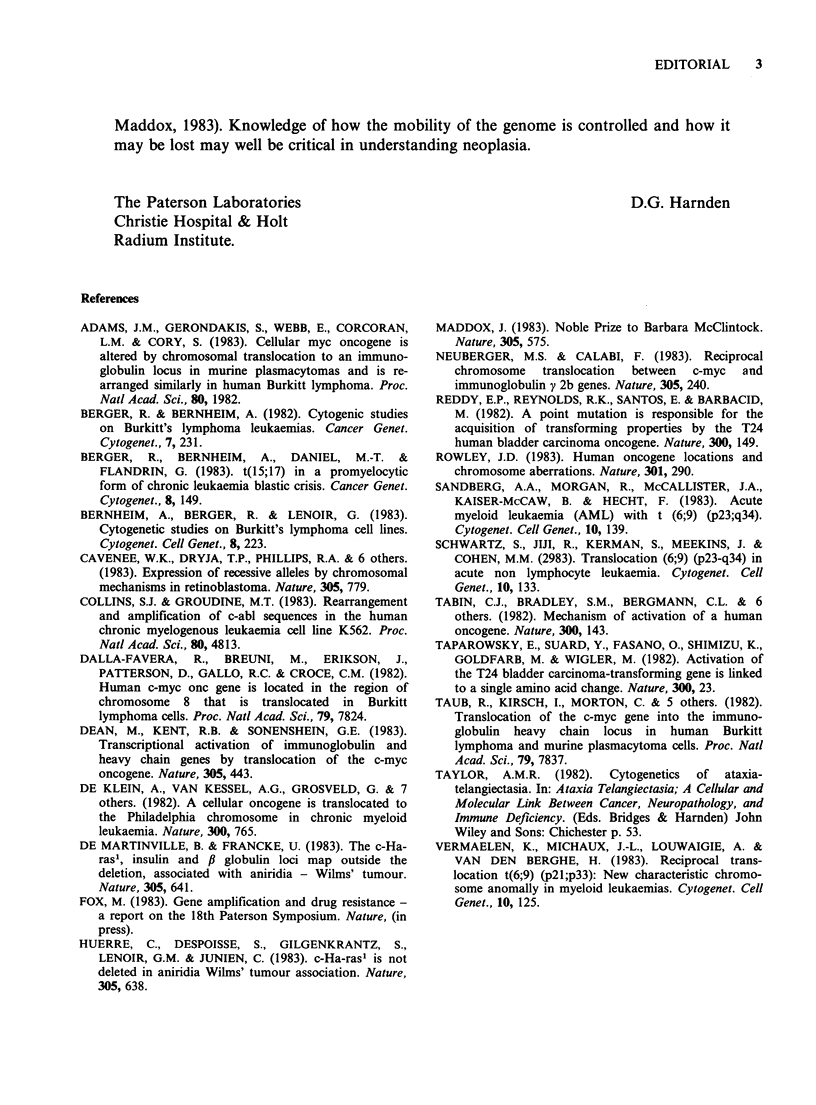# Genetic change in the cancer cell.

**DOI:** 10.1038/bjc.1984.1

**Published:** 1984-01

**Authors:** D. G. Harnden


*Br. J. Cancer* (1984), **49**, 1–3

### Editorial

### Genetic change in the cancer cell

The association of the chromosomal location of cellular proto-oncogenes with the exchange points involved in specific chromosomal rearrangements observed in particular human neoplasms has drawn attention once again to the role of alterations in the cellular genome in the initiation and maintenance of neoplasia. The recent observations have not only shed new light on old problems, they have also led to a more critical appraisal of the available data with some intriguing results. It seems likely that even a detailed understanding of these associations may not fully explain the loss of growth control and the aberrant cell behaviour observed in cancer.

There are now several examples of the close association of specific chromosomal exchange points in human neoplasms with the location of cellular proto-oncogenes (summarized by Rowley, 1983). The classic translocation between chromosomes 9 and 22 in chronic granulocytic leukaemia (CGL) involves a breakpoint on chromosome 9 close to the c-abl gene which is translocated to the abnormal chromosome 22 (the Philadelphia chromosome) (de Klein *et al.*, 1982). There is now some evidence of abnormal expression of the translocated c-abl gene in a CGL derived cell line (Collins & Groudine, 1983). It is hard to avoid the conclusion, because of the specificity of the rearrangement, that the c-abl oncogene is in some way involved in the abnormal proliferation of the myeloid cells in CGL. More direct evidence of involvement of a cellular proto-oncogene comes from the case of the t(8; 14) translocation in B cell lymphomas (Dalla-Favera *et al.*, 1982). In that case it is clear that the c-myc gene and the immunoglobulin (Ig) genes are directly invloved in the exchanges (Taub *et al.*, 1982; Adams *et al.*, 1983). The argument for a causal relationship is strengthened by the closely analogous mouse model where junction fragments between a proto-oncogene and the Ig genes have been recognized, e.g. the translocation between c-myc and the $C_\alpha$ region of IgA and IgG producing mouse myeloma lines (Dean *et al.*, 1983) or between c-myc and both $C_{\gamma 2b}$ and $V_H$ genes in another myeloma line (Neuberger & Calabi, 1983).

If these arrangements are a vital part of oncogenesis why is it that they appear to occur in only a minority of neoplasms and then not in all cases nor in all cells? Part of the answer is certainly that we have not looked in the right way nor thoroughly enough. Recent reports suggest that specific translocations involving chromosome 8 along with chromosomes 14, 2 or 22 do occur in all Burkitt tumours if adequately studied (Berger & Bernheim, 1982; Bernheim *et al.*, 1983). Similarly, the paradox of the specific t(15; 17) translocations occurring in only some cells of some cases of acute promyelocytic leukaemia seems to have been explained by the observation by Berger *et al.* (1983) that slight differences in technique may favour the recognition of chromosomally normal erythroblasts at the expense of the translocation carrying leukaemic promyelocytes. New specific aberrations are being discovered regularly such as the t(6;9) translocation which appears to define a specific sub-group of acute myeloid leukaemias (Vermaelen *et al.*, 1983; Schwartz *et al.*, 1983; Sandberg *et al.*, 1983).

Other classes of genomic change not overtly connected with chromosomal change are being recognized. The single base change at position 35, in the c-ras$^H$ gene recovered by transfection of DNA from the T24 human bladder carcinoma line into NIH 3T3 cells provides the first direct evidence of the involvement of point mutation in human neoplasia (Reddy *et al.*, 1982; Tabin *et al.*, 1982; Taparowsky *et al.*, 1982). The guanosine to thymidine mutation which changes amino acid 12 from glycine to valine confers on the gene product transforming properties not possessed by the unaltered gene product. It appears that this change is a dominant expression of the altered gene product, but without suitable gene markers in the original cell and since DNA mediated transformation is only possible in already abnormal cell lines such as NIH 3T3 we cannot be absolutely sure of this.

Deletion or chromosome loss also seem possible routes to the expression of the malignant phenotype. Specific chromosomal deletions have been associated for some time with a proportion of cases of Wilms' tumour and of retinoblastoma. More specific evidence comes from the recent observation by Cavenee *et al.* (1983) that in retinoblastoma patients heterozygous for variants of esterase D (an enzyme closely linked to the retinoblastoma locus) and for restriction site polymorphisms on chromosome 13, the cells of the tumour express only one allele, strongly suggesting a deletion in, or complete loss of one chromosome 13 and it is tempting to speculate that this hemizygosity permits expression of a gene on the homologous chromosome in some way associated with the malignant behaviour. The danger of jumping to conclusions is, however, demonstrated by the recent observation that the c-ras$^{H}$ gene lies outside the segment of chromosome 11 deleted in Wilms' aniridia patients (Huerre *et al.*, 1983; de Martinville & Francke, 1983).

There is thus mounting evidence that these very specific genomic changes are part of the neoplastic process. However, there is also evidence to suggest that while they may be essential they may not be sufficient to lead to frank neoplasia. For example, translocation of the c-abl and c-cis genes in t(9; 22) exchange is not sufficient to give loss of cellular control in the erythroid cell lineage in CGL patients. Similarly in ataxia telangiectasia specific translocations involving 14q12 are associated with clonal proliferation of lymphocytes without overall loss of control on white cell numbers (Taylor, 1982). Something more catastrophic must occur before uncontrolled neoplasia arises.

Most advanced tumours have grossly abnormal chromosomes with loss, gain and rearrangement. Second, gene amplification (which appears vital in developing drug resistance in some cancers) is exceptionally difficult to induce in normal cells but easy to induce in undifferentiated tumour cells; tumour cells showing some degree of differentiation can be induced to undergo gene amplification only with difficulty (Fox, 1983). This could suggest that the more advanced malignant phenotype is accompanied by a very significant loosening of the controls on the precision of DNA replication and of chromosomal segregation. It seems possible that early and highly specific changes be they exchange, point mutation or deletion may lead directly or indirectly to a breakdown in the precise controls and that this may in turn lead on to neoplasia.

We have now become familiar with the concept of the fluidity of the genotype of the somatic cell in both a functional and evolutionary sense. In the former case the generation of antibody diversity involves major genomic rearrangements while antigenic changes in some lower organisms are determined by the movement of genes or groups of genes into expression loci. In an evolutionary sense we have evidence of gene amplification followed by modification as well as the derivation of pseudogenes via RNA intermediates. It is pleasing therefore that the role of Barbara McClintock in having the audacity to challenge the idea of the static genome over forty years ago should now be acknowledged by the award of a Nobel Prize (an appreciation by J.

Maddox, 1983). Knowledge of how the mobility of the genome is controlled and how it may be lost may well be critical in understanding neoplasia.

The Paterson Laboratories
Christie Hospital & Holt
Radium Institute.

D.G. Harnden

**References**

ADAMS, J.M., GERONDAKIS, S., WEBB, E., CORCORAN, L.M. & CORY, S. (1983). Cellular myc oncogene is altered by chromosomal translocation to an immunoglobulin locus in murine plasmacytomas and is rearranged similarly in human Burkitt lymphoma. *Proc. Natl Acad. Sci.*, **80**, 1982.

BERGER, R. & BERNHEIM, A. (1982). Cytogenic studies on Burkitt's lymphoma leukaemias. *Cancer Genet. Cytogenet.*, **7**, 231.

BERGER, R., BERNHEIM, A., DANIEL, M.-T. & FLANDRIN, G. (1983). t(15;17) in a promyelocytic form of chronic leukaemia blastic crisis. *Cancer Genet. Cytogenet.*, **8**, 149.

BERNHEIM, A., BERGER, R. & LENOIR, G. (1983). Cytogenetic studies on Burkitt's lymphoma cell lines. *Cytogenet. Cell Genet.*, **8**, 223.

CAVENEE, W.K., DRYJA, T.P., PHILLIPS, R.A. & 6 others. (1983). Expression of recessive alleles by chromosomal mechanisms in retinoblastoma. *Nature*, **305**, 779.

COLLINS, S.J. & GROUDINE, M.T. (1983). Rearrangement and amplification of c-abl sequences in the human chronic myelogenous leukaemia cell line K562. *Proc. Natl Acad. Sci.*, **80**, 4813.

DALLA-FAVERA, R., BREUNI, M., ERIKSON, J., PATTERSON, D., GALLO, R.C. & CROCE, C.M. (1982). Human c-myc onc gene is located in the region of chromosome 8 that is translocated in Burkitt lymphoma cells. *Proc. Natl Acad. Sci.*, **79**, 7824.

DEAN, M., KENT, R.B. & SONENSHEIN, G.E. (1983). Transcriptional activation of immunoglobulin and heavy chain genes by translocation of the c-myc oncogene. *Nature*, **305**, 443.

DE KLEIN, A., VAN KESSEL, A.G., GROSVELD, G. & 7 others. (1982). A cellular oncogene is translocated to the Philadelphia chromosome in chronic myeloid leukaemia. *Nature*, **300**, 765.

DE MARTINVILLE, B. & FRANCKE, U. (1983). The c-Ha-ras[1], insulin and $\beta$ globulin loci map outside the deletion, associated with aniridia – Wilms' tumour. *Nature*, **305**, 641.

FOX, M. (1983). Gene amplification and drug resistance – a report on the 18th Paterson Symposium. *Nature*, (in press).

HUERRE, C., DESPOISSE, S., GILGENKRANTZ, S., LENOIR, G.M. & JUNIEN, C. (1983). c-Ha-ras[1] is not deleted in aniridia Wilms' tumour association. *Nature*, **305**, 638.

MADDOX, J. (1983). Noble Prize to Barbara McClintock. *Nature*, **305**, 575.

NEUBERGER, M.S. & CALABI, F. (1983). Reciprocal chromosome translocation between c-myc and immunoglobulin $\gamma$ 2b genes. *Nature*, **305**, 240.

REDDY, E.P., REYNOLDS, R.K., SANTOS, E. & BARBACID, M. (1982). A point mutation is responsible for the acquisition of transforming properties by the T24 human bladder carcinoma oncogene. *Nature*, **300**, 149.

ROWLEY, J.D. (1983). Human oncogene locations and chromosome aberrations. *Nature*, **301**, 290.

SANDBERG, A.A., MORGAN, R., McCALLISTER, J.A., KAISER-McCAW, B. & HECHT, F. (1983). Acute myeloid leukaemia (AML) with t (6;9) (p23;q34). *Cytogenet. Cell Genet.*, **10**, 139.

SCHWARTZ, S., JIJI, R., KERMAN, S., MEEKINS, J. & COHEN, M.M. (2983). Translocation (6;9) (p23-q34) in acute non lymphocyte leukaemia. *Cytogenet. Cell Genet.*, **10**, 133.

TABIN, C.J., BRADLEY, S.M., BERGMANN, C.L. & 6 others. (1982). Mechanism of activation of a human oncogene. *Nature*, **300**, 143.

TAPAROWSKY, E., SUARD, Y., FASANO, O., SHIMIZU, K., GOLDFARB, M. & WIGLER, M. (1982). Activation of the T24 bladder carcinoma-transforming gene is linked to a single amino acid change. *Nature*, **300**, 23.

TAUB, R., KIRSCH, I., MORTON, C. & 5 others. (1982). Translocation of the c-myc gene into the immunoglobulin heavy chain locus in human Burkitt lymphoma and murine plasmacytoma cells. *Proc. Natl Acad. Sci.*, **79**, 7837.

TAYLOR, A.M.R. (1982). Cytogenetics of ataxia-telangiectasia. In: *Ataxia Telangiectasia; A Cellular and Molecular Link Between Cancer, Neuropathology, and Immune Deficiency*. (Eds. Bridges & Harnden) John Wiley and Sons: Chichester p. 53.

VERMAELEN, K., MICHAUX, J.-L., LOUWAIGIE, A. & VAN DEN BERGHE, H. (1983). Reciprocal translocation t(6;9) (p21;p33): New characteristic chromosome anomally in myeloid leukaemias. *Cytogenet. Cell Genet.*, **10**, 125.